# Problem gamblers share deficits in impulsive decision-making with alcohol-dependent individuals

**DOI:** 10.1111/j.1360-0443.2009.02533.x

**Published:** 2009-06

**Authors:** Andrew J Lawrence, Jason Luty, Nadine A Bogdan, Barbara J Sahakian, Luke Clark

**Affiliations:** 1 Behavioural and Clinical Neuroscience Institute, University of CambridgeCambridge, UK; 2 Department of Experimental Psychology, University of CambridgeCambridge, UK; 3 Southend Community Drug and Alcohol Service, South Essex Partnership NHS TrustEssex, UK; 4 Derwent Centre, North Essex Partnership Foundation TrustHarlow, Essex, UK; 5 Department of Psychiatry, University of Cambridge School of Clinical MedicineCambridge, UK

**Keywords:** Addiction, alcohol, decision-making, impulsivity, pathological gambling, prefrontal cortex, risk-taking, vulnerability

## Abstract

**Aims:**

Problem gambling has been proposed to represent a ‘behavioural addiction’ that may provide key insights into vulnerability mechanisms underlying addiction in brains that are not affected by the damaging effects of drugs. Our aim was to investigate the neurocognitive profile of problem gambling in comparison with alcohol dependence. We reasoned that shared deficits across the two conditions may reflect underlying vulnerability mechanisms, whereas impairments specific to alcohol dependence may reflect cumulative effects of alcohol consumption.

**Design:**

Cross-sectional study.

**Setting:**

Out-patient addiction treatment centres and university behavioural testing facilities.

**Participants:**

A naturalistic sample of 21 male problem and pathological gamblers, 21 male alcohol-dependent out-patients and 21 healthy male control participants.

**Measurements:**

Neurocognitive battery assessing decision-making, impulsivity and working memory.

**Findings:**

The problem gamblers and alcohol-dependent groups displayed impairments in risky decision-making and cognitive impulsivity relative to controls. Working memory deficits and slowed deliberation times were specific to the alcohol-dependent group.

**Conclusions:**

Gambling and alcohol-dependent groups shared deficits in tasks linked to ventral prefrontal cortical dysfunction. Tasks loading on dorsolateral prefrontal cortex were selectively impaired in the alcohol-dependent group, presumably as a consequence of long-term alcohol use.

## INTRODUCTION

Gambling is a common recreational activity in which approximately 70% of the British population engage at least annually [1,2], but which becomes dysfunctional in a minority. Problem gambling, where the behaviour has a negative impact on everyday function (e.g. debt, interpersonal conflict), has a prevalence of 1–4% in western populations [3,4], whereas the more stringent DSM-IV-TR diagnosis of ‘pathological gambling’ has a prevalence of 0.5–1.5% [5]. In the DSM-IV-TR [6], pathological gambling is classified as an impulse control disorder (ICD). However, the diagnostic criteria are modelled on those for substance dependence, emphasizing the negative impact of symptoms on social and personal function. Pathological gambling shares clinical features with substance dependence, with evidence of cravings [7], withdrawal [8,9] and tolerance [10]. There is considerable comorbidity with substance use disorders [11], and common genetic risk factors have been implicated [12]. This aetiological overlap may also be present for milder problem gambling [13,14]. These findings have led to the suggestion that problem and pathological gambling may be conceptualized as a ‘behavioural addiction’[15–17], or part of an ‘addiction syndrome’[18], which may share vulnerability mechanisms with substance use disorders but, crucially, in the absence of harmful consequences of chronic drug administration.

By this account, we would predict overlap between problem gambling and substance use disorders in their neurocognitive profiles. Predisposing factors associated with the vulnerability to addiction should be present in both problem gamblers and substance users. Impairments associated with the long-term progressive effects of drug use should be present in substance users but absent in problem gamblers. The primary objective of the present study was to test this prediction in an exploratory assessment of neurocognitive function in problem gamblers, alcohol-dependent individuals and healthy controls.

We were interested specifically in a set of higher-level cognitive control processes associated with the dorsal and ventral sectors of the prefrontal cortex (PFC). We hypothesized that impairments in executive function (digit span, spatial working memory), linked to the integrity of dorsolateral PFC, would be restricted to the alcohol-dependent group [19–21], arising as a consequence of long-term alcohol consumption. In contrast, we predicted that risky decision-making and reflection impulsivity would be abnormal in both problem gamblers and individuals with alcohol dependence, reflecting vulnerability mechanisms in ventral fronto-striatal circuitry [12,22]. Recent research has begun to characterize changes in impulsivity and decision-making in treatment-seeking pathological gamblers [12,23–26]. The present study used two tasks, the Cambridge Gamble Task and the Information Sampling Task, which have not been studied previously in problem gambling or alcohol dependence. Gamblers in the present study were non-treatment-seeking and recruited through advertisements, and comprised a mixture of problem [South Oaks Gambling Screen (SOGS) ≥ 3] and probable pathological gamblers (SOGS ≥ 5).

## METHODS

### Participants

Problem gamblers (*n* = 21; mean age: 37.0 ± 9.6 years) were recruited through community advertisements and the GamCare website (http://www.gamcare.org.uk). All respondents were male. All gamblers scored ≥ 3 on the SOGS [27], indicative of problem gambling, and 15 respondents (71%) met the more stringent criteria for probable pathological gambling (SOGS ≥ 5); we refer to this combined group henceforth as ‘problem gamblers’.

Alcohol-dependent subjects (*n* = 21; all male; SOGS ≤ 2) were out-patients at drug and alcohol treatment centres (Southend Community Drug and Alcohol Service, Essex, UK; Cambridge Drug and Alcohol Service, Cambridge, UK). Diagnosis of alcohol dependence was confirmed using DSM-IV-TR criteria in a semi-structured interview by a psychiatrist (J.L./N.B.). Sobriety at time of testing was confirmed by breath alcohol readings ≤ 0.01 mg/l (Lion Alcometer S-D2; Lion Laboratories Ltd, Barry, UK). Four subjects had consumed alcohol in the past 48 hours, and all others were abstinent for >1 week, with 12 subjects meeting criteria for remission. The mean self-reported duration of abstinence was 150 days (239 days in those meeting remission criteria). Eight subjects were receiving medication (disulfiram: 4, antidepressants: 6, benzodiazepines: 2).

Exclusion criteria, assessed by means of a locally developed screening tool, were: age over 65 years, comorbid psychiatric illness (with the exception of depression in the alcohol-dependent group), history of head injury or neurological disorder. Healthy controls (*n* = 21; all male; SOGS ≤ 2) were recruited through community advertisements and from a panel of research volunteers.

### Procedure

The protocol was approved by the Cambridge Local Research Ethics Committee (03/313 and 05/Q0108/286) and all volunteers provided written informed consent. All subjects completed the SOGS [27] to index problematic gambling behaviour, the Beck Depression Inventory (BDI) version 2 [28] to measure depressive symptoms and the 10-item Drug Abuse Screening Test (DAST-10) [29] to index use of illicit drugs. The alcohol-dependent group completed the Severity of Alcohol Dependence Questionnaire (SADQ) [30]; problem gamblers and controls completed the three-item Alcohol Use Disorders Identification Test (AUDIT-C) [31] to indicate alcohol consumption.

The neurocognitive assessment comprised the following measures (see Supporting Information; details at the end of this paper):

1. *Cambridge Gamble Test (CGT) [32]*: a test of decision-making under risk. On each trial, the subject is presented with an array of 10 boxes coloured red and blue, in varying ratios of red : blue boxes. The subject is required to make a probability judgement (which colour hides a concealed token) followed by a wager. Dependent measures were decision-making quality (the proportion of trials where the majority colour was selected), decision-making latency (average response time to make the probability decision) and the average percentage bet. Bankruptcies (where subjects lost all points within a block) were also analysed.
2. *Information Sampling Test (IST) [33]*: a test of ‘reflection’ impulsivity, measuring the tendency to gather and evaluate information prior to making a decision. Subjects can sample information by opening boxes from a grid and must decide which colour is in the majority. Dependent variables were the probability of making the correct decision given the information sampled (P_(correct)_) and the number of incorrect decisions (errors). The average number of boxes opened is also reported.
3. *CANTAB Spatial Working Memory (SWM) [34]*: a self-ordered search task requiring monitoring of spatial information in working memory. Dependent measures were total between-search errors (opening a box that has previously yielded a token) and strategy score.
4. *Digit Span (forwards/backwards)* from the Wechsler Adult Intelligence Scale [35] was used to index the maintenance and manipulation of verbal information in working memory.

There were missing values for a small number of subjects on SWM (three controls) and Digit Span (two alcohol-dependent, one control) due to time constraints.

### Statistical analysis

Normally distributed data were analysed with analysis of variance (ANOVA), using Greenhouse–Geisser's epsilon where sphericity assumptions were violated. Between-group comparisons were investigated *post-hoc* using Fisher's least significant differences protected *t*-test. Non-parametric data were analysed with Kruskal–Wallis rank-transform tests or χ^2^ tests. Correlations were assessed using Kendall's Tau-B concordance tests. As an exploratory study, all tests were thresholded at *P* < 0.05, two-tailed, with no correction for multiple comparisons.

## RESULTS

### Demographics and clinical questionnaires

Demographic and clinical data are reported in Table 1. There were no significant differences in age or years of education. The mean SOGS score for the gamblers was 9.7 [standard deviation (SD) 5.8], consistent with mean scores in previous studies (mean 9.4–12.6) [36–41]. The mean SADQ for the alcohol-dependent group was 33.7 (SD 16.0), which is indicative of severe alcohol dependence (>30) and consistent with mean scores for previous studies (mean 26.9–34.2) [42–44]. Alcohol consumption (AUDIT-C) was greater in problem gamblers than controls (*F*_(1,40)_ = 7.30, *P* = 0.01). Illicit drug use (DAST-10) was more common in the problem gamblers than controls (χ^2^ = 6.46, df 1, *P* = 0.011), with a marginally significant difference between the alcohol-dependent group and controls (χ^2^ = 2.79, df 1, *P* = 0.095). Depressed mood was increased in the problem gamblers and alcohol-dependent groups relative to controls (*F*_(2,59)_ = 14.88, *P* < 0.001), and in the alcohol-dependent group compared to the problem gamblers (*P* = 0.009) (Table 1).

**Table 1:** Demographic and clinical data.

	*PG (n* = *21)*	*AD (n* = *21)*	*HC (n* = *21)*	*Test statistic*	*Post-hoc effects of group*
Age	37.0 ± 9.6	44.2 ± 9.2	40.2 ± 13.6	*F*_(2,60)_ = 2.23, NS	–
Years of education	12.9 ± 2.9	11.9 ± 3.4	13.5 ± 2.4	*F*_(2,60)_ = 1.70, NS	–
South Oaks Gambling Screen Score	9.67 ± 5.8	0.57 ± 0.87	0.24 ± 0.54	–	–
SADQ—Alcohol Dependence Severity	–	33.7 ± 16.0	–	–	–
AUDIT-C Alcohol Consumption	7.1 ± 3.0	–	4.7 ± 2.7	*F*_(1,40)_ = 7.30, *P* = 0.011	–
Drug Abuse Screening Test^a^	2.5 ± 2.8	1.8 ± 2.9	0.5 ± 1.3	χ^2^_(2)_ = 7.16, *P* = 0.028	PG>HC
Beck Depression Inventory II	13.2 ± 9.7	20.9 ± 11.8	5.3 ± 4.3	*F*_(2,59)_ = 14.88, *P* < 0.001	AD>PG>HC

PG: problem gambler; AD: alcohol-dependent; HC: healthy control.

a Data distributed non-normally, tested using Kruskal–Wallis; NS: not significant.

### Neuropsychological tasks

#### Cambridge Gamble Task

Bankruptcies were significantly more common in problem gamblers (*n* = 5, 24%; Fisher's exact test, *P* = 0.048), and approached significance in the alcohol-dependent group (*n* = 4, 19%; Fisher's exact test, *P* = 0.11) when compared with controls (*n* = 0). Mixed-model ANOVAs of decision-making quality and decision latency were conducted, with one within-subjects factor (box ratio; 9 : 1, 8 : 2, 7 : 3, 6 : 4) and one between-subjects factor (group). For the analysis of decision-making quality, there was a significant main effect of box ratio (*F*_(1.9,112.4)_ = 11.0, *P* = 0.001), such that subjects were more likely to choose the colour in the majority at higher ratios. The main effect of group was not significant (*F*_(1,60)_ = 1.08, *P* = 0.34), nor the group × box ratio interaction (*F*_(3.7,112.4)_ = 1.49, *P* = 0.21). For decision latency, there were significant main effects of box ratio (*F*_(2.6,146.0)_ = 5.41, *P* = 0.003), as subjects tended to deliberate longer when the ratio was less certain. There was a significant main effect of group (*F*_(2,57)_ = 5.14, *P* = 0.009), but no group × box ratio interaction (*F*_(5.1,146.0)_ = 1.53, *P* = 0.18). *Post-hoc* investigation of the main effect of group revealed slower decision-making in the alcohol-dependent group compared to controls (*P* = 0.003) and problem gamblers (*P* = 0.035), with no difference between the problem gamblers and controls (*P* = 0.41) (Table 2).

**Table 2:** Neuropsychological test performance.

	*PG (n* = *21)*^a^	*AD (n* = *21)*^a^	*HC (n* = *21)*^a^	*Test statistic*	*Post-hoc effects of group*
Cambridge Gamble Task					
Total points obtained	1772 ± 1205	1605 ± 805	1551 ± 592	*F*_(2,60)_ = 0.34, NS	–
Bankruptcies^b^	5 (24%)	4 (19%)	0 (0%)	χ^2^(1) = 5.25, *P* = 0.022	–
% Rational decisions	90 ± 19	94 ± 9	96 ± 9	*F*_(2,60)_ = 1.15, NS	–
Percentage wager	59 ± 17	56 ± 11	48 ± 13	*F*_(2,56)_ = 3.31, *P* = 0.045	PG>HC
Decision latency (ms)	2064 ± 739	2742 ± 1136	1970 ± 753	*F*_(2,56)_ = 5.74, *P* = 0.005	AD>[PG=HC]
Information Sampling Task					
Errors	5.0 ± 3.2	4.8 ± 2.5	3.2 ± 2.4	*F*_(2,60)_ = 2.41, *P* = 0.099	PG>HC
Boxes opened (/25)	8.6 ± 3.6	9.8 ± 4.1	12.8 ± 4.6	*F*_(2,60)_ = 5.92, *P* = 0.005	[PG=AD]<HC
Spatial working memory					
Total errors	23.3 ± 22.8	40.3 ± 30.0	22.8 ± 21.4	*F*_(2,57)_ = 3.20, *P* = 0.048	AD>[PG=HC]
Strategy	32.0 ± 6.2	31.8 ± 8.5	29.5 ± 5.4	*F*_(2,57)_ = 0.73, NS	–
Digit span					
Forwards score (/12)	9.7 ± 1.8	8.3 ± 1.9	10.1 ± 1.8	*F*_(2,57)_ = 5.30, *P* = 0.008	AD<[PG=HC]
Backwards score (/12)	8.6 ± 3.0	6.5 ± 2.2	7.8 ± 2.5	*F*_(2,57)_ = 3.51, *P* = 0.037	AD<PG

PG: problem gambler; AD: alcohol-dependent; HC: healthy control.

a Sample size reduced for some tests as specified in the Results text.

b Indicates the number of individuals with at least one bankruptcy.

PG and AD groups collapsed to ensure validity of χ^2^ test; NS: not significant.

**Figure 1:**
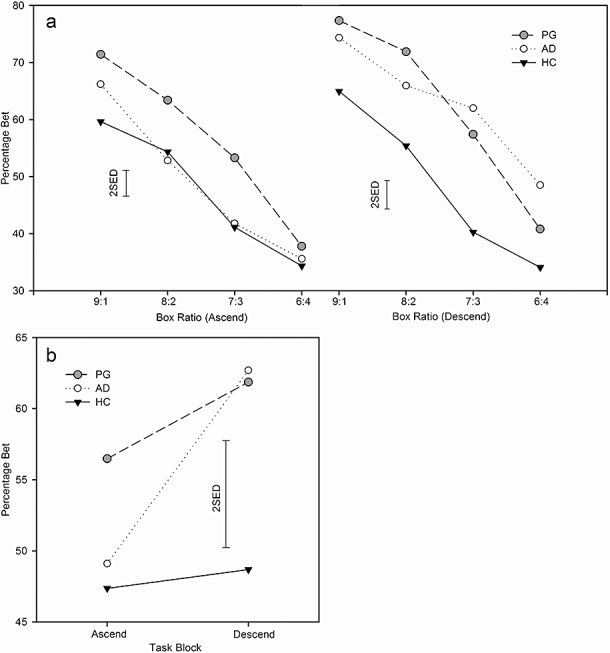
Wagering on the Cambridge Gamble Task was elevated in both alcohol-dependent (AD) and problem gambler (PG) groups, compared to healthy controls (HC). (a) Problem gamblers placed higher bets than healthy controls regardless of task condition or box ratio. (b) Betting behaviour in the ascending and descending conditions, collapsed across box ratios. Alcohol-dependent subjects placed higher bets, particularly in the descend condition. SED: standard error of the difference after Cardinal & Aitken (2006 [67]) p. 98 [SED = √(2MS_error_/n^h^) where n^h^ is the harmonic mean of the group sizes]

Betting behaviour was analysed with a mixed-model ANOVA with factors of group, box ratio, an additional within-subjects factor of condition (ascend, descend) and an additional between-subjects factor of condition order. The effects including condition order did not approach significance (*P* > 0.25), and condition order was therefore excluded from the model. The main effects of box ratio (*F*_(2.1,116.3)_ = 100.2, *P* < 0.001), condition (*F*_(1,56)_ = 10.10, *P* = 0.003) and group (*F*_(2,56)_ = 3.45, *P* = 0.039) reached significance in the presence of a significant condition × ratio × group three-way interaction (*F*_(6,168)_ = 2.37, *P* = 0.035). The condition × group interaction also approached significance (*F*_(2,56)_ = 3.08, *P* = 0.055); other two-way interaction terms were non-significant. To explore the three-way interaction (see Fig. 1), pairwise ANOVAs were conducted. These ANOVAs confirmed the significant main effect of box ratio, indicating adjustment of betting by the changing odds. In the comparison of problem gamblers against controls, the main effect of group was significant (*F*_(1,36)_ = 5.17, *P* = 0.029), but the interactions group × condition (*F*_(1,36)_ = 0.92, *P* = 0.34) and group × box ratio × condition (*F*_(3,108)_ = 0.715, *P* = 0.55) were not. Thus, wagering in the problem gamblers was elevated relative to controls regardless of task condition, and the two groups showed similar risk adjustment. In the comparison of the alcohol-dependent group against controls, the main effects of group (*F*_(1,40)_ = 4.60, *P* = 0.038) and condition (*F*_(1,40)_ = 9.01, *P* = 0.005) reached significance as well as the condition × group (*F*_(1,40)_ = 6.10, *P* = 0.018) and condition × box ratio × group (*F*_(3,130)_ = 3.60, *P* = 0.016) interactions. The alcohol-dependent and control groups did not differ in their wagering in the ascend condition (group: *F*_(1,40)_ = 0.185, *P* = 0.6; box ratio × group: *F*_(2.3,91.4)_ = 1.39, *P* = 0.25) but diverged in the descend condition, where alcohol-dependent subjects placed higher wagers (group: *F*_(1,40)_ = 8.51, *P* = 0.006). This condition × group interaction was particularly strong in trials with unfavourable odds (6 : 4 box ratio: *F*_(1,40)_ = 5.73, *P* = 0.021; 7 : 3 box ratio: *F*_(1,40)_ = 11.34, *P* = 0.002) rather than favourable odds (9 : 1 box ratio: *F*_(1,40)_ = 0.29, *P* = 0.59; 8 : 2 box ratio: *F*_(1,40)_ = 2.97, *P* = 0.092), indicating that the group difference was strongest at the lower odds. When problem gambler and alcohol-dependent groups were compared directly, no effects in the ANOVA model reached statistical significance apart from the main effect of box ratio.

### Information sampling test

**Figure 2:**
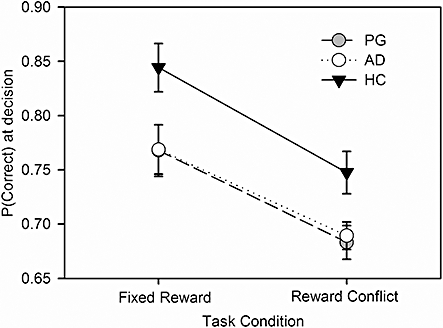
Reflection impulsivity on the Information Sampling Task. Problem gamblers (PG) and alcohol-dependent (AD) groups sampled less information than healthy controls (HC), responding at a lower certainty of being correct. *P*_(correct)_ calculated using the formula:

*P*_(correct)_ data were analysed using a mixed-model ANOVA of condition (fixed reward, reward conflict) × group. There was a significant main effect of condition (*F*_(1,60)_ = 44.17, *P* <0.001), due to subjects sampling less information in the reward conflict condition compared to the fixed reward condition. There was a significant main effect of group (*F*_(2,60)_ = 4.76, *P* = 0.013), but no group × condition interaction (*F*_(2,60)_ = 0.45, *P* = 0.63). *Post-hoc* tests (collapsed across condition) found that, compared to controls, both alcohol-dependent (*P* = 0.025) and problem gambler (*P* = 0.005) groups tolerated significantly more uncertainty in their decisions. In keeping with this reduction in reflection, there was also a trend towards a between-group difference in error rates (*F*_(2,60)_ = 4.75, *P* = 0.099) with a significant difference *post-hoc* between gamblers and controls (*P* = 0.045) and a trend level difference between alcohol-dependents and controls (*P* = 0.093) (Fig. 2).

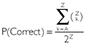

where *Z* = 25 (number of boxes opened) and A = 13 (number of boxes of the chosen colour). Error bars represent ±1 standard error of the mean

### Spatial working memory

**Figure 3:**
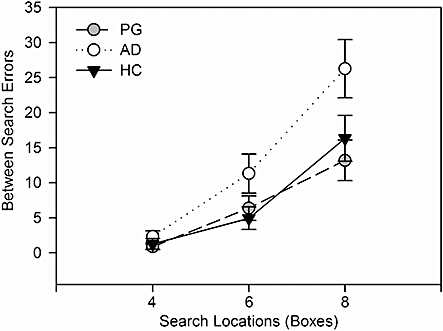
Spatial Working Memory Task performance was impaired in the alcohol-dependent (AD) group compared to problem gamblers (PG) and healthy controls (HC). Between-search errors are responses to box locations that have previously yielded tokens. Error bars represent ±1 standard error of the mean

Between-search errors were analysed using a mixed-model ANOVA of difficulty (within-subjects) × group (between-subjects). Main effects of difficulty (*F*_(1.4,82.5)_ = 79.3, *P* < 0.001) and group (*F*_(2,57)_ = 3.85, *P* = 0.027) were observed along with a significant difficulty × group interaction (*F*_(1.4,82.3)_ = 3.90, *P* = 0.013) (see Fig. 3). Simple main-effects analysis at each level of difficulty revealed a significant group difference at the eight-box level (*F*_(2,57)_ = 4.58, *P* = 0.015), where alcohol-dependent subjects made more errors than both controls (*P* = 0.037) and problem gamblers (*P* = 0.005). Controls and problem gamblers did not differ at the eight-box stage (*P* = 0.51) or at the other levels. There was no significant difference in strategy score (*F*_(2,57)_ = 0.74, *P* = 0.48).

### Digit span

Scores were analysed using a mixed-model ANOVA of condition (forwards, backwards) × group. All groups performed more poorly in the backwards condition (*F*_(1,57)_ = 43.7, *P* < 0.001), and there was also a significant main effect of group (*F*_(2,57)_ = 4.71, *P* = 0.013), with no significant interaction term (*F*_(2,57)_ = 1.84, *P* = 0.16). Between-group comparisons revealed poorer digit span performance in the alcohol-dependent group compared with problem gamblers (*P* = 0.006) and controls (*P* = 0.017), with no difference between problem gamblers and controls (*P* = 0.73).

## DISCUSSION

This study found neurocognitive deficits present in both alcohol dependence and problem gambling, relative to healthy controls who were group-matched for age and education. While some deficits were common to the two target groups, others were unique to the alcohol-dependent group. Specifically, there were shared neuropsychological impairments in reflection impulsivity (Information Sampling Test) and risky decision-making (Cambridge Gamble Task). In contrast, the alcohol-dependent group showed significant deficits in working memory (digit span, CANTAB Spatial Working Memory) and decision-making deliberation times (Cambridge Gamble Task latencies), compared to both the controls and problem gambler groups.

Problem gambling has been described as a prototypical model of addiction that is not confounded by the direct damaging effects of substances of abuse [15,11]. Neurocognitive and neurobiological investigation of problem gamblers may therefore provide insight into the underlying vulnerability mechanisms across the addictions. Our findings of overlapping impairments in decision-making and reflection impulsivity in the two target groups adds to a growing body of data that implicate these processes as pre-existing vulnerability factors in the addictions [12,22]. For example, two prospective studies have reported that trait impulsivity predicts later problem gambling [13,45], as well as alcohol, cannabis and nicotine dependence [45], at a 3-year follow-up. Neurocognitive deficits in impulse control and decision-making have been shown to predict treatment outcomes in substance users [46] and pathological gamblers [47], suggesting that psychological rehabilitation of impulsive decision-making may have clinical benefits. The problem gamblers in the present study reported more alcohol use (AUDIT), drug use (DAST-10) and depression (BDI-II) compared to controls. This is consistent with widely observed clinical comorbidities in problem gambling [5,48]. The neurocognitive variables that differed between problem gamblers and controls were not associated significantly with these clinical scores (see Supporting Information Table S1, details at the end of this paper), suggesting that they do not explain the deficits observed. Nevertheless, it seems likely that elevated alcohol and drug consumption, even at levels below the criteria for abuse or dependence, may impact adversely upon cognitive function in gamblers, and this merits consideration in further research.

The Cambridge Gamble Task was administered as a test of risky decision-making, with some ecological validity to real-life gambling behaviour. In comparison to the widely used Iowa Gambling Task, the Cambridge Gamble Task is a test of decision-making under risk (i.e. with explicit probabilities) rather than ambiguity. The task also minimizes demands for learning, working memory and cognitive flexibility, which complicate the interpretation of Iowa Gambling Task effects [49]. On the Cambridge Gamble Task, the alcohol-dependent and problem gambler groups displayed significant increases in their betting behaviour relative to controls, with no significant difference between the two target groups. This pattern of responding is highly reminiscent of previous data in patients with focal lesions to the ventromedial PFC [50]. Indeed, other studies have indicated ventromedial PFC pathophysiology in treatment-seeking pathological gamblers [26,51,52] and alcohol dependence [53–55] using the Iowa Gambling Task. Blaszczynski & Nower's [56] pathways model proposes three aetiologically distinct subgroups of problem gamblers, with neuropsychological impairment linked to the most extreme ‘antisocial impulsivist’ gamblers. Our findings extend the earlier reports, but are less compatible with the pathways model, by demonstrating neurocognitive sequelae in less severe problem gamblers recruited through community advertising rather than a treatment service. It is evident, however, that risky decision-making is a quantitative effect in both the gamblers and alcohol-dependent subjects (see Supporting Information Fig. S1, details at the end of this paper), rather than a categorical deficit, and that these deficits may be aligned with sources of heterogeneity in problem gambling.

A common deficit was also observed on the Information Sampling Task, which assesses the tendency to gather and evaluate information before reaching a decision (see also Kagan [57]). Both target groups opened fewer boxes and tolerated more uncertainty in their decisions than the controls. As a probable consequence of reduced information sampling, the gamblers and alcohol-dependent groups showed a (non-significant) tendency to make more errors on the task, which represents the hallmark of this subtype of impulsivity [58]. We have shown previously similar changes in reflection impulsivity in current and former users of amphetamine and opiates [33], as well as regular cannabis users [59]. The current data extend these findings to alcohol dependence as well as a putative behavioural addiction (problem gambling), which supports the role of reflection impulsivity as a potential vulnerability marker in the addictions. Deficits in reflection impulsivity may also be associated with ventromedial PFC pathology, as patients with ventromedial PFC lesions displayed impaired reflection on another widely used test of this construct, the Matching Familiar Figures Test [57,60].

The two measures that detected group differences in the problem gamblers both involved abstract points reinforcement, and some degree of resemblance to real-life gambling. While it is conceivable that the gamblers' performance may have been affected by their extensive experience with large monetary wins and probabilistic games, as well as the cognitive distortions that tend to accompany gambling [61], the presence of these deficits in the alcohol-dependent group mitigates against such an explanation. Moreover, recent functional imaging data suggest that cognitive features linked to problem gambling, including loss-chasing and the effects of near-miss outcomes, may also be mediated by brain networks involving the ventromedial PFC [62,63].

While cognitive impulsivity was present in both groups, working memory was selectively impaired in the alcohol-dependent group. These subjects committed more errors on both spatial working memory and a test of verbal working memory (Digit Span). The former deficit entailed excessive returns to locations where tokens had been found previously, and was more evident at the harder levels of the task. Executive dysfunction is a common finding in alcohol-dependent populations [19,64]. Moreover, our finding of unimpaired working memory in problem gamblers is consistent with two previous reports using self-ordered pointing tasks [39,41]. We interpret the dissociation between the two groups as consistent with the long-term consequences of alcohol exposure on dorsolateral PFC function [19–21]. Chronic alcohol administration may be associated with cell death or tissue shrinkage in this area [65]. The alcohol-dependent group also displayed slowed decision-making, consistent with psychomotor slowing [35]. However, from the current cross-sectional findings we cannot exclude the possibility that alcohol dependence is associated with a selective pre-existing deficit in dorsolateral PFC that is absent in problem gamblers. It is also possible that depressive symptoms or medication may have contributed to the executive deficits in the alcohol-dependent group, although an effect of depression is unlikely given the lack of correlations against BDI score.

A number of limitations should be noted. As an exploratory investigation, group sizes were small and statistical analyses were not corrected for multiple comparisons. Further research is required with larger groups to confirm these commonalities and differences in neurocognitive function across problem gambling and alcohol dependence. While our alcohol-dependent subjects were recruited through specialist clinics, no equivalent facilities existed for the recruitment of problem gamblers, who were therefore recruited through community and internet advertising. Clinician-confirmed diagnoses were not available for the problem gamblers, who were identified instead by a widely used and extensively validated self-report measure (SOGS ≥ 3). Common comorbidities were tolerated, as exclusion would lead to a highly unrepresentative sample [5]; depression, alcohol consumption and drug use were measured using self-report scales and correlated against neurocognitive indices. We did not assess nicotine use systematically, although some of the subjects smoked and were permitted to take breaks during testing if required. Previous research has reported elevated impulsivity in smokers [66], and future research is needed to confirm that the common deficits in gamblers and alcohol dependence are not explained by smoking behaviour. We also acknowledge that problem gambling is a heterogeneous condition, and preferred forms of gambling (e.g. horse-racing versus slot machine play) may conceivably impact upon neurocognitive performance.

In summary, the present data indicate overlapping impairments in reflection impulsivity and risky decision-making in a community-recruited group of problem gamblers and a clinically referred group of alcohol-dependent patients. This profile is consistent with pathophysiology in the ventromedial PFC, and the presence of this profile in a putative behavioural addiction supports impulsive decision-making as a candidate vulnerability marker in the addictions. The alcohol-dependent participants showed additional neurocognitive deficits in working memory and deliberation, which are hypothesized to reflect long-term effects of alcohol consumption on the dorsal PFC.

## Supporting information

**Figure S1** Individual datapoints for amount bet on the Cambridge Gamble Task.

**Table S1** Results of correlational analysis of neurocognitive performance against clinical questionnaires.

**Appendix S1** Full description of neurocognitive tests and results of correlational analysis of neurocognitive performance against clinical questionnaires.
