# Supplementary material for: Problem gamblers share deficits in impulsive decision-making with alcohol-dependent individuals
Source: Addiction. 2009 Jun;104(6):1006–15. doi: 10.1111/j.1360-0443.2009.02533.x (PMC2773538; doi:10.1111/j.1360-0443.2009.02533.x)
Supplement: Supplementary file 1 [file add0104-1006-SD1.doc]

***Figure S1 Individual datapoints for amount bet on the Cambridge Gamble Task***
