# Supplementary material for: Problem gamblers share deficits in impulsive decision-making with alcohol-dependent individuals
Source: Addiction. 2009 Jun;104(6):1006–15. doi: 10.1111/j.1360-0443.2009.02533.x (PMC2773538; doi:10.1111/j.1360-0443.2009.02533.x)
Supplement: Supplementary file 2 [file add0104-1006-SD2.doc]

***Table S1 Results of correlational analysis of neurocognitive performance against clinical questionnaires***

|  |  | Neurocognitive Variables | | | | | |
| --- | --- | --- | --- | --- | --- | --- | --- |
|  |  | CG Percent Bet | CG  Decision Latency | IST P(correct)  Total | SWM  Total Errors | DS Forward | DS Backward |
| PG | SOGS | -0.046 | - | -0.292 | - | - | - |
|  | AUDIT-C | 0.047 | - | 0.237 | - | - | - |
|  | DAST-10 | -0.056 | - | 0.054 | - | - | - |
|  | BDI-II | -0.074 | - | -0.173 | - | - | - |
| AD | SADQ | -0.014 | 0.248 | -0.295 | 0.053 | -0.106 | -0.063 |
|  | Abstinence | 0.034 | 0.000 | -0.211 | **0.427** | -0.162 | -0.019 |
|  | BDI-II | **0.379** | 0.160 | 0.074 | 0.219 | 0.038 | 0.076 |
|  | DAST | 0.275 | -0.315 | 0.000 | 0.181 | 0.054 | -0.131 |

Results of Kendall’s Tau-B measure of concordance. Tau values in **boldface** indicate significance at p<0.05 level. PG - Problem Gambler, AD - Alcohol Dependent. CG – Cambridge Gamble Task, IST – Information Sampling Task, SWM – Spatial Working Memory task, DS – Digit Span. SOGS – South Oaks Gambling Screen score, AUDIT-C – Alcohol Use Disorders Identification Test Consumption measure, DAST – Drug Abuse Screening Test, BDI – Beck Depression Inventory, SADQ – Severity of Alcohol Dependence Questionnaire, Abstinence – time since last consumption of alcohol.
