# Supplementary material for: Problem gamblers share deficits in impulsive decision-making with alcohol-dependent individuals
Source: Addiction. 2009 Jun;104(6):1006–15. doi: 10.1111/j.1360-0443.2009.02533.x (PMC2773538; doi:10.1111/j.1360-0443.2009.02533.x)
Supplement: Supplementary file 3 [file add0104-1006-SD3.doc]

**Supporting Information**

***Appendix S1 Full description of neurocognitive tests and results of correlational analysis of neurocognitive performance against clinical questionnaires***

***Methods***

Subjects were screened for exclusion criteria by means of a locally-developed screening questionnaire that asked about current medications, previous head injury (resulting in >30 seconds unconsciousness), presence of any (lifetime) psychiatric or neurological conditions, and whether the subject had ever visited a mental health professional. Comorbid psychiatric conditions that did not involve referral to a mental health profession and/or drug treatment were did not warrant exclusion; rather, the established co-morbidities (drug use, alcohol consumption, depression) were measured in a continuous manner and regressed against neurocognitive variables of interest. In the alcohol and gambling groups, current depression was not considered an exclusion factor (where depression onset occurred after the onset of alcohol/gambling problems) as this condition is commonly comorbid and would result in over-selection of the sample.

The neurocognitive assessment was completed in a quiet testing room, using an Advantech touch-sensitive PC. Forwards and backwards digit span from the Wechsler Adult Intelligence Scale [1] were used to index the maintenance and manipulation of information in working memory. Details on the further tests is provided below:

***Cambridge Gamble Test (CGT) [2]***

The CGT is a test of decision-making under risk. The subject is presented with a row of 10 boxes, coloured red or blue and is informed that a yellow token has been hidden at random in one of the boxes. Their first decision is to indicate whether the token will be under a red or blue box, by pressing one of two panels labelled RED and BLUE at the foot of the display. The ratio of coloured boxes varies across trials from 9:1 to 5:5. The subject is then asked to wager some points on the outcome of their red-blue decision. Wagers are offered in points values representing 5, 25, 50, 75 and 95% of the total, increasing or decreasing every 2s. The subject presses the bet box to place the currently displayed wager. Subjects complete 2 blocks in the Ascend condition, where the bet is initially low (5%) as increases over time, and 2 blocks in the Descend condition, where the bet is initially high (95%) and decreases over time [3]. After placing the wager, the token is revealed and bet is added or subtracted to the points total, depending on the outcome. Each block comprises 9 trials, where the points total is reset to 100 at the start of the block. Condition (Ascend, Descend) order is counterbalanced between subjects. Three dependent measures were extracted: 1) decision-making quality is the proportion of trials where the majority colour was selected, 2) decision-making latency is the average response time to make the red-blue decision, 3) betting behaviour is analysed as the average percentage bet for trials where the box colour in the majority was selected. Ambiguous (5:5) trials were excluded from all analyses. Bankruptcies were also recorded, where the points score drops below 1 during one block. In some cases, bankruptcy can produce missing data for a subject in some cells. To make best use of data, cases with missing variables were excluded according to the variable being used, i.e. decision-making quality data were analysed for subjects missing betting data. In this report, one gambler was missing all data and a further three were missing data for latency and betting measures. Repeating these analyses with exclusion of partial cases listwise did not alter the findings.

***Information Sampling Test (IST) [4]***

The IST is a test of ‘reflection’ impulsivity, assessing the tendency to gather and evaluate information prior to making a decision. On each trial, a 5x5 grid of covered boxes is presented above two coloured panels. Touching a covered box immediately reveals one of the two colours presented beneath. Subjects were instructed to decide which colour was in the majority, and told “It is entirely up to you how many boxes you open before making your decision”. Responses were made by pressing the appropriate coloured box beneath the grid. Once opened, boxes remained visible for the duration of the trial to minimize working memory demands. Trials completed in less than 30 seconds resulted in a variable inter-trial interval to limit the minimum trial length to 30 seconds, to avoid rapid responding due to delay-aversion. Subjects completed one practice trial followed by 10 trials in each of two conditions. In one condition (Fixed Reward), the reward obtained for a correct decision (100 points) was independent of the amount of information sampled. In the other condition (Reward Conflict), sampling more information resulted in a smaller reward (an initial reward of 250 points reduced by 10 points for each box opened). Incorrect decisions resulted in a fixed loss of 100 points. The order of the two conditions was counterbalanced between subjects. Dependent variables were the probability of making the correct decision given the current ratio of coloured boxes (P_(Correct)_), and the number of incorrect decisions. The number of boxes opened is also reported in Table 2; this measure is highly correlated with P_(correct)_. However, if 14 boxes are opened, they may be distributed between 7:7 (hence P_(correct)_=0.5) through to 14:0 (hence P_(correct)_ =1.0), and hence P_(correct)_ is considered a superior measure of the information sampled.

***CANTAB Spatial Working Memory (SWM) [5]***

SWM is a self-ordered spatial search task where the subject is presented with an array of boxes, and is required to search the boxes for hidden tokens. Each box in the array will only yield a token once, so that searches can be progressively restricted to boxes that are yet to hold a token. This requires the monitoring and updating of information in working memory. Subjects complete 4 practice trials with 3 boxes, followed by 4 trials each at three levels of difficulty (4, 6 and 8 boxes). Dependent measures are the number of Between-Search Errors (opening a box that has previously yielded a token) and a Strategy Score. An effective strategy for this task is to commence each search from the same box. The Strategy Score is calculated from the number of searches starting from unique boxes for 6 and 8 box trials, thus a lower score indicates better strategy use.

***Statistical Analysis***

Analyses were conducted using Statistical Package for the Social Sciences v15.0.1.1 (SPSS; Chicago, Illinois). Proportion data were arcsine transformed and latency data were log_10_-transformed to improve suitability for parametric statistics (tables and figures present untransformed scores). Normally distributed data were analyzed with analysis of variance (ANOVA), with Greenhouse-Geisser’s epsilon where sphericity assumptions were violated. All relevant factors were included in initial models, but, where appropriate, terms which contributed insignificantly (main effects and interactions with alpha>0.25) were excluded to increase power [6]. Between-group comparisons were investigated *post-hoc* using Fisher’s Least Significant Differences protected t-test. Categorical data were tested using Chi-squared tests, collapsing categories where >20% of cells had expected frequencies less than 5 or any below 1. Non-parametric continuous data were analyzed with Kruskal-Wallis rank-transform tests. *A-priori* hypotheses regarding variable associations were investigated using Kendall’s Tau-b coefficients within each group, to minimize the influence of outliers on the small sample sizes [7].

***Results***

In Supplementary Figure 1 we present the distribution of scores on the Cambridge Gamble Task betting measure, in relation to the overall group means (horizontal lines). Problem gamblers are subdivided into those meeting criteria for probable Pathological Gambling (SOGS≥5; N=15, open circles) and those meeting the less stringent problem gambler criteria (SOGS≥3; N=5, grey filled circles). These data indicate that the between-group differences in risky decision-making are a quantitative, continuous difference rather than categorical or bimodal. Moreover, the effect in the problem gamblers is representative for individuals meeting criteria for both problem gambling and the more stringent probable Pathological Gambling.

***Correlations between Neurocognitive and Clinical Variables***

The severity of addiction in the two groups (measured by SOGS in the problem gamblers, and SADQ in the alcohol dependent group) was correlated against task variables where those groups differed significantly from controls. No significant associations were observed (see Supplementary Table 1). In the alcohol dependent group, neurocognitive variables were correlated with the length of abstinence from alcohol (log transformed), to examine associations with recency of use. It should be noted that duration of abstinence was assessed by self-report. Whilst there was a significant association between SWM between-search errors and abstinence (Tau_(19)_ = 0.43, p=0.007), the direction of this effect (longer abstinence associated with worse performance) is inconsistent with an effect caused by recent or acute alcohol use. There were no further associations found with abstinence (Tau = 0.00 – 0.21; P = 1.00 – 0.18).

To assess the validity of the drug free model, measures of alcohol and drug use (AUDIT-C & DAST-10) in the problem gamblers were correlated with the two neurocognitive variables that discriminated gamblers and controls (CGT-betting and IST-P_(correct)_). There were no significant associations with drug or alcohol use (Tau= -0.056 – 0.237, p>.155).

Correlations between neurocognitive performance and BDI-II score were conducted in the problem gamblers and alcohol dependent groups, given between group differences in depressive symptoms. No significant associations were present in the gamblers (Tau = 0.074 – 0.173; p = 0.276 – 0.680). In the alcohol dependent group, depression was significantly associated with percentage bet on the CGT (Tau_(18)_=0.379, p=0.021).

***References***

1. Lezak, M., Howieson, D. & Loring, D. *Neuropsychological Assessment* Oxford: Oxford University Press; 2004.

2. Rogers, R. D., Everitt, B. J., Baldacchino, A., Blackshaw, A. J., Swainson, R., Wynne, K. et al. Dissociable deficits in the decision-making cognition of chronic amphetamine abusers, opiate abusers, patients with focal damage to prefrontal cortex, and tryptophan-depleted normal volunteers: evidence for monoaminergic mechanisms, *Neuropsychopharmacology* 1999; 20: 322-39.

3. Miller, L. A. Impulsivity, risk-taking, and the ability to synthesize fragmented information after frontal lobectomy, Neuropsychologia 1992; 30: 69-79.

4. Clark, L., Robbins, T. W., Ersche, K. D. & Sahakian, B. J. Reflection impulsivity in current and former substance users, Biol Psychiatry 2006; 60: 515-22.

5. Owen, A. M., Downes, J. J., Sahakian, B. J., Polkey, C. E. & Robbins, T. W. Planning and spatial working memory following frontal lobe lesions in man, Neuropsychologia 1990; 28: 1021-34.

6. Cardinal, R. N. & Aitken, M. R. F. ANOVA for the Behavioural Sciences Researcher. New Jersey: Lawrence Erlbaum Associates; 2006.

7. Kruskal, W. H. Ordinal Measures of Association, Journal of the American Statistical Association 1958; 53: 814-861.
